# High-quality chromosome-scale de novo assembly of the *Paspalum notatum* ‘Flugge’ genome

**DOI:** 10.1186/s12864-022-08489-6

**Published:** 2022-04-11

**Authors:** Zhenfei Yan, Huancheng Liu, Yu Chen, Juan Sun, Lichao Ma, Aihua Wang, Fuhong Miao, Lili Cong, Hui Song, Xue Yin, Qi Wang, Yayun Gong, Guofeng Yang, Zengyu Wang

**Affiliations:** 1 College of Grassland Science, Qingdao Agricultural University, Qingdao, 266109 China; 2 Key Laboratory of National Forestry and Grassland Administration on Grassland Resources and Ecology in the Yellow River Delta, Qingdao Agricultural University, Qingdao, 266109 China; 3 College of Animal Science, Qingdao Agricultural University, Qingdao, 266109 China; 4 Berry Genomics Corporation, Beijing, China

**Keywords:** *Paspalum notatum* ‘Flugge’, Genome, De novo assembly, Genome annotation

## Abstract

**Background:**

*Paspalum notatum* ‘Flugge’ is a diploid with 20 chromosomes (2n = 20) multi-purpose subtropical herb native to South America and has a high ecological significance. It is currently widely planted in tropical and subtropical regions. Despite the gene pool of *P. notatum* ‘Flugge’ being unearthed to a large extent in the past decade, no details about the genomic information of relevant species in Paspalum have been reported. In this study, the complete genome information of *P. notatum* was established and annotated through sequencing and de novo assembly of its genome.

**Results:**

The latest PacBio third-generation HiFi assembly and sequencing revealed that the genome size of *P. notatum* ‘Flugge’ is 541 M. The assembly result is the higher index among the genomes of the gramineous family published so far, with a contig N50 = 52Mbp, scaffold N50 = 49Mbp, and BUSCOs = 98.1%, accounting for 98.5% of the estimated genome. Genome annotation revealed 36,511 high-confidence gene models, thus providing an important resource for future molecular breeding and evolutionary research. A comparison of the genome annotation results of *P. notatum* ‘Flugge’ with other closely related species revealed that it had a close relationship with *Zea mays* but not close compared to *Brachypodium distachyon*, *Setaria viridis, Oryza sativa*, *Puccinellia tenuiflora*, *Echinochloa crusgalli*. An analysis of the expansion and contraction of gene families suggested that *P. notatum* ‘Flugge’ contains gene families associated with environmental resistance, increased reproductive ability, and molecular evolution, which explained its excellent agronomic traits.

**Conclusion:**

This study is the first to report the high-quality chromosome-scale-based genome of *P. notatum* ‘Flugge’ assembled using the latest PacBio third-generation HiFi sequencing reads. The study provides an excellent genetic resource bank for gramineous crops and invaluable perspectives regarding the evolution of gramineous plants.

**Supplementary Information:**

The online version contains supplementary material available at 10.1186/s12864-022-08489-6.

## Background

*Paspalum notatum* (*P. notatum*) ‘Flugge’ is a subtropical grass native to South America belonging to the Poaceae family [1], including diploid and apomictic polyploid biotypes [2]. It has excellent agronomic traits such as fast growth, strong reproductive ability, and resistance to cold, barrenness, high temperature, submergence, and erosion [3–6]. It has been used for water and soil conservation, environmental protection, ecological restoration, and landscaping, among other uses, thus greatly improving people’s lives amongst other economic and ecological benefits [7, 8]. The grass does not have strict soil type requirements and possesses a strong ability to grow on sandy soils with lower fertility and aridity [9]. These advantages make it the commonly used turfgrass during the warm seasons [10]. It provides a huge feeding value for the livestock industry [11, 12], thus necessitating increased planting in recent years. The grass is widely planted in tropical and subtropical regions. Notably, different environments provide a new source of genetic novelty for *P. notatum* ‘Flugge’, making it grow rapidly and adapt to various environmental changes [13–15].

*P. notatum* ‘Flugge’ as a very important subtropical grass but its biological researches, especially at the genomic level, is far less than other members of gramineous plants [16–18], such as *Sorghum bicolor* and *Zea mays*, and the *Setaria viridis* family. Though it is an important sub-family in the millet tribe, the Paspalinae, there are only a few reports about its genome [19, 20]. Only the reference genome of *E. crusgalli* in the genus Echinochloa has been reported [21]. The lack of this information restricts our understanding of the evolutionary history of Paspalum and the ability to fully tap the genetic potential of this species for breeding superior varieties, especially in the context of global climate changes [22, 23].

Mapping the genome differences between *P. notatum* ‘Flugge’ and its related species using a robust phylogenetic framework is a basis for a comprehensive understanding of the evolution of its genes and genomes [24, 25]. Moreover, using Hi-C technology to observe the collinearity between the chromosomes of *P. notatum* ‘Flugge’ and its related species significantly improves the accuracy and sensitivity of gene evolution research and enables the prediction of more robust genome structure patterns [26, 27]. Using the complete genomics information of *P. notatum* ‘Flugge’ and that of the closely related species for biological analysis can provide many valuable contributions to the analysis of the differentiation and evolution mechanism of the Poaceae Paspalum. In this study, we obtained the genome information of *P. notatum* ‘Flugge’ by combing Illumina, Pacbio HiFi, and Hi-C to fully understand its genome content and molecular evolution history. The study also aimed to identify the historical events and continuous changes of the geographical environment, the positive selection of genes, and the systematic evolution of *P. notatum* ‘Flugge’. The study provides a starting point for evolutionary genomics studies and a new research direction for analyzing the evolutionary relationships between *P. notatum* ‘Flugge’ and its related species [28, 29].

## Results

### Genome-survey, sequencing, and assembly

**Fig. 1:**
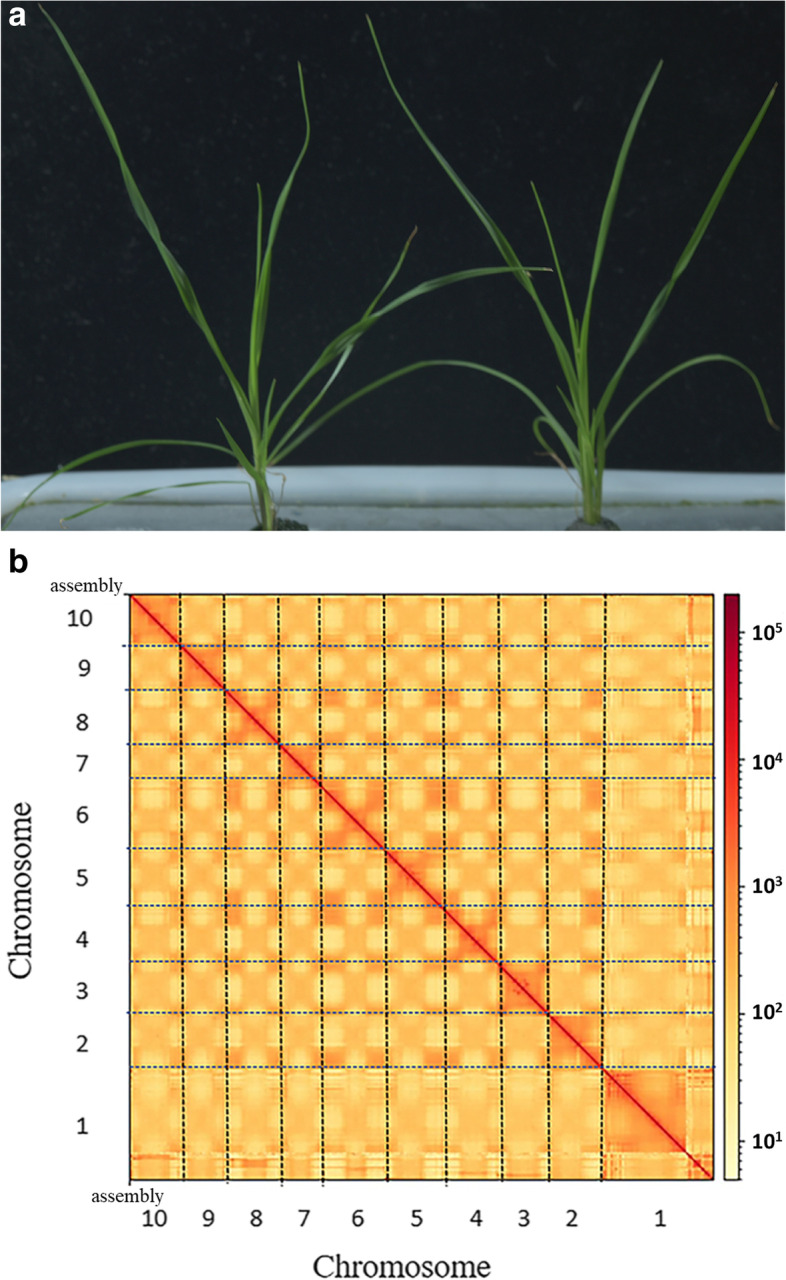
Plant morphology and Hi-C-assisted genome assembly of *P. notatum* ‘Flugge’. **a** Phenotype of the sequenced *P. notatum* ‘Flugge’ plant. **b** Hi-C interaction heatmap showing 100-kb resolution super scaffolds

This study evaluated the genome size, repeatability, heterozygosity, and other genome parameters of *P. notatum* ‘Flugge’ (Fig. 1a) [30]. Quality control results of the offline data revealed 57Gbp of Illumina data, with a GC content of 46.08%. A comparison of 10,000 randomly selected clean reads to the NT library through blasting revealed a 96.12% mapping. K-mer analysis performed to estimate the complexity of the genome further predicted a genome size of 549 M, with 1.16% of repeat sequence and 58.33% of heterozygous sequence.

The genome sequence of *P. notatum* ‘Flugge’ was predicted using the traditional second-generation sequencing (NGS) data assembly method and the third generation HiFi sequencing (Third-Generation Sequencing, TGS) developed by PacBio [31]. Besides TGS making up for some of the shortcomings of NGS in assembly applications, it also did not require PCR amplification, produced ultra-long read lengths, and had no GC preference. Therefore, using PacBio HiFi for genome assembly is an effective assembly strategy. High-quality HiFi reads were obtained after parameter comparison of the output data. The HiFi reads were 1.9Mbp, with an N50 measure of 1.4kbp.

The contigs were subsequently generated based on the phased string graph [32]. The assembled genome (541 M) contained 79 contigs, with an N50 of 52Mbp and a maximum contig size of 125Mbp. The average GC content of the assembled genome was 45.65% (Table 1), which was higher than that of *Oryza sativa* (43.65%) [30] and *Cynodon transvaalensis* (43.6%) [32]. The Illumina reads were subsequently compared with the DNA library to evaluate the quality and completeness of the assembly. The comparison yielded 93.77% of the properly mapped reads. Moreover, the single-copy orthologous gene library used to evaluate the completeness of the genetic space revealed a BUSCO [33] of 98.1% of the assembled genome, highlighting that it had good integrity.

**Table 1:** Summary statistic for the *Paspalum notatum* ‘Flugge’ genome

	Assembly	
Genome assembly	Estimated genome size	549 M
	Total length of assembly	541 M
	Number of contigs	79
	Contig N50	52Mbp
	Largest contig	126Mbp
	Number of scaffolds	49
	Scaffold N50	49Mbp
	Chromosome coverage(%)	95.15%
	GC content of genome	45.65%
	Annotation	Total length
Transposable elements	Total	328Mbp(60.64%)
	Retrotransposon	263Mbp(49.38%)
	DNA Transposon	20Mbp (3.71%)
		Copies
Noncoding RNAs	rRNAs	828
	tRNAs	846
	miRNAs	133
	snRNAs	1708
Gene models	Number of genes	36,511
	Mean gene length	4029 bp
	Mean coding sequence length	1503 bp

### Scaffold construction and curation

Hi-C is a high-throughput chromosome conformation capture technology. It utilizes the entire cell nucleus as the research object, fixes and captures the mutual sites in the chromosomes, and then performs high-throughput sequencing to study the spatial distribution of chromatin DNA in the whole genome [34, 35]. A high-resolution chromatin regulatory element interaction map is obtained from the positional relationship. In this study, we generated chromosome-level super scaffolds using the Hi-C data with 60G and genome coverage of 110X. Subsequent analysis of the results of the Hi-C library revealed a genome with a genome size of 540 Mbp and a scaffold N50 of 49 Mbp. Moreover, 514 Mb genome sequences were mapped to 10 chromosomes after Hi-C assisted assembly, accounting for 95.15% of the sequences. The linkages between and within chromosomes was calculated upon completion of the Hi-C assisted assembly to further verify the accuracy of the assembly results. The linkages within the chromosomes were much stronger than between the chromosomes. Moreover, the linkages of chromosomes in a close physical location were much stronger than in a distant physical location (Fig. 1b). These findings suggested that the assembly result was correct. Table 1 summarizes the assembly information.

### Genome annotation

The gene functions in the genome are inferred by calculating the homology alignments and predicting its repetitive sequences. In this study, we identified MITEs repetitive sequences and LTR transposable elements, accounting for 60.64 and 46.61% of the total sequence, respectively, using structure prediction methods. The LTR-retrotransposons of Copia and Gypsy was 10.03 and 25.31%, respectively. In addition, there were 2827 single repeats identified in the assembled genome. There were 12 types of ncRNA totaling 3907 ncRNA.

In the same line, 36,511 high-confidence gene models were obtained using RNA-seq and de novo prediction strategies after eliminating gene models containing premature stop codons and frameshifts. The gene models were unevenly distributed on ten chromosomes.

**Fig. 2:**
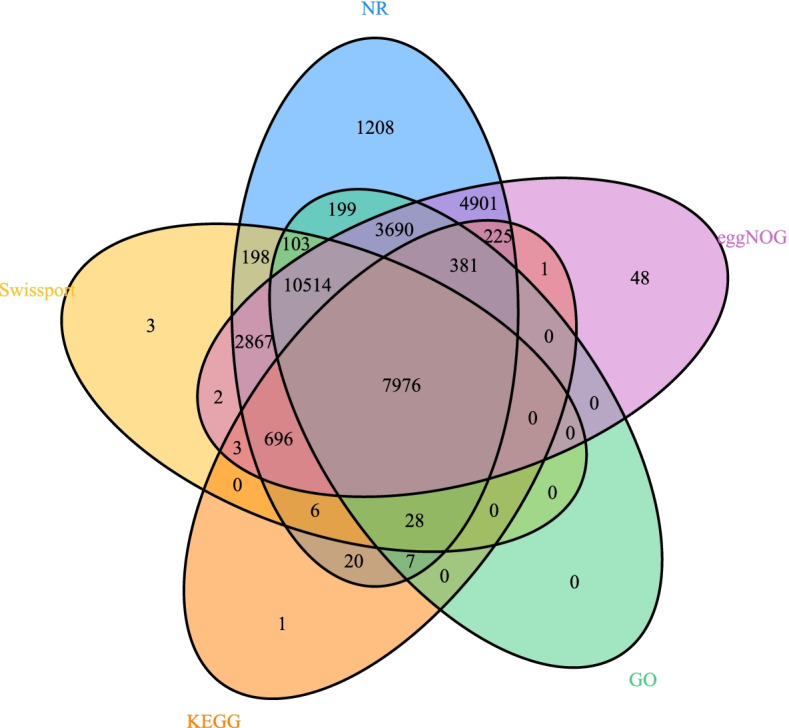
Venn analysis of five major databases(NR, Swiss-Prot, eggNOG, GO, KEGG) containing gene function annotation information

The average gene length was 4029 bp, with each gene containing an average of five exons. The average lengths of CDS, exons, and introns were 1503 bp, 319 bp, and 599 bp, respectively. We also compared *Paspalum notatum* ‘Flugge’ with five related species, including *Puccinellia tenuiflora*, *Zea mays*, *Sorghum bicolor*, *Echinochloa c*, *Echinochloa h*, and *Brachypodium distachyon*. *Zea mays* had the largest genome (~ 2.1GB) [36] that was 3.8 times that of *Paspalum notatum* ‘Flugge’. An assembly of *Echinochloa C* with the largest number of genes (103853), *Brachypodium distachyon,* which had the smallest number of genes (30002), and the other four species which had similar numbers of genes, revealed similar average CDS lengths (Table 2). Annotation comparison analysis of five databases used to annotate the genomes annotated 22,900 genes and predicted the functions of different genes and the number and proportion of genes corresponding to them and revealing a data set of 7976 known common genes (Fig. 2).

**Table 2:** The information of annotated gene models per species for all the species

Organism	Number of genes	Mean CDS length(bp)	Exons per transcript	Mean exon length(bp)	Mean intro length(bp)
*Paspalum notatum‘Flugge’*	36,511	1503	5.0	319	599
*Puccinellia tenuiflora*	38,387	1281	5.9	284	455
*Zea mays*	44,717	1379	8.9	327	486
*Sorghum bicolor*	31,705	1403	7.6	341	340
*Echinochloa C*	103,853	1272	5.2	244	421
*Echinochloa H*	37,510	1278	5.1	250	440
*Brachypodium distachyon*	30,002	1435	7.9	346	264

### Gene family and evolution analysis

**Fig. 3:**
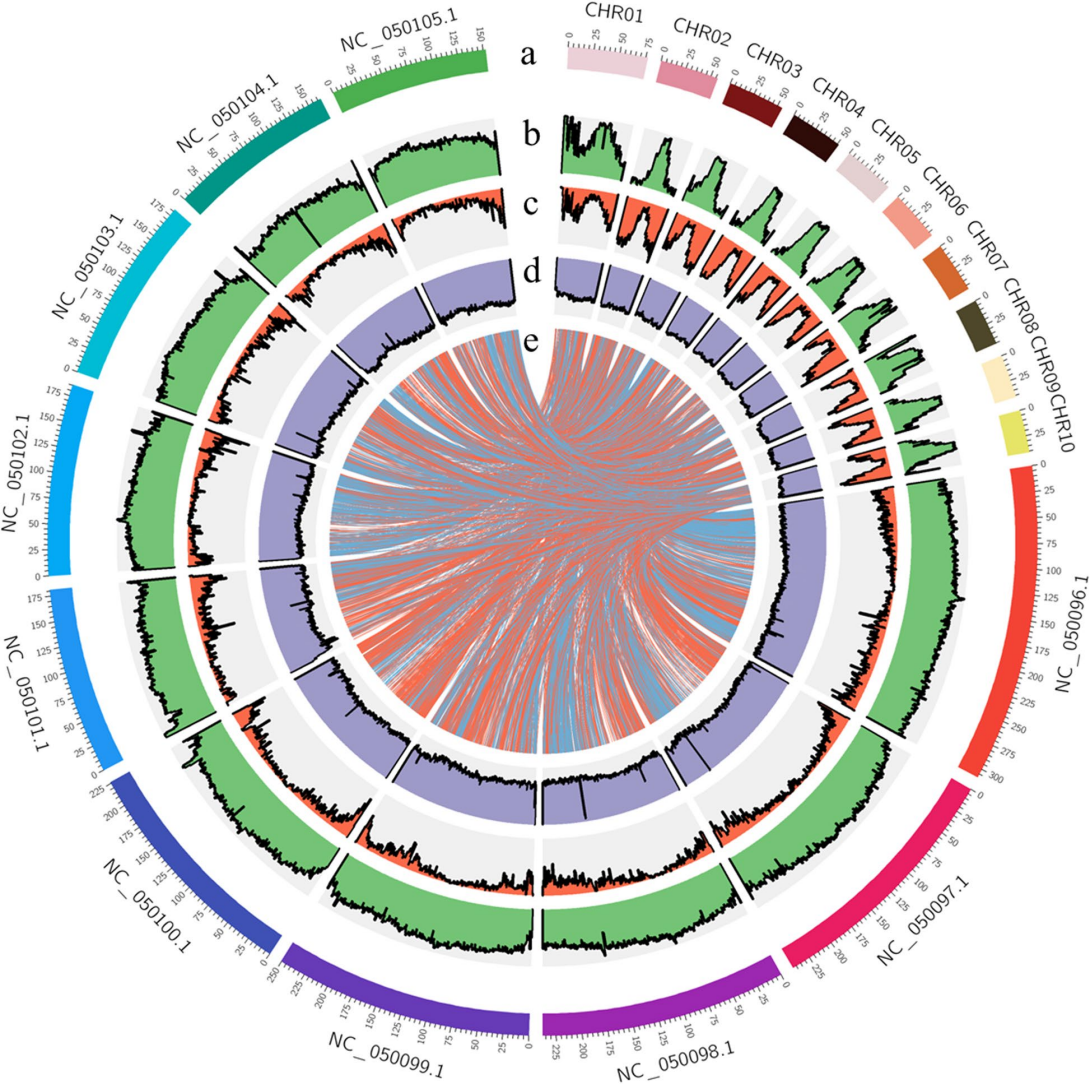
Features of the *P. notatum* ‘Flugge’ and *Z. mays* genome. **a** Length of each pseudochromosome (Mb). **b** Distribution of repetitive sequence. **c** Distribution of gene density. **d** Distribution of the GC content (**e**) *P. notatum* ‘Flugge’ and *Zea mays* synteny analysis; the beginning of NC represents the chromosome of *Zea mays,* while the beginning of CHR represents the chromosome of *P. notatum* ‘Flugge’

Collinearity analysis suggested that the chromosomes of *P. notatum* ‘Flugge’ and *Zea mays* showed a certain degree of synchronization. The ten chromosomes of *P. notatum* ‘Flugge’ and ten of *Zea mays* had a good collinear relationship (Fig. 3), indicating that the chromosomes were conserved after the differentiation of the two species.

**Fig. 4:**
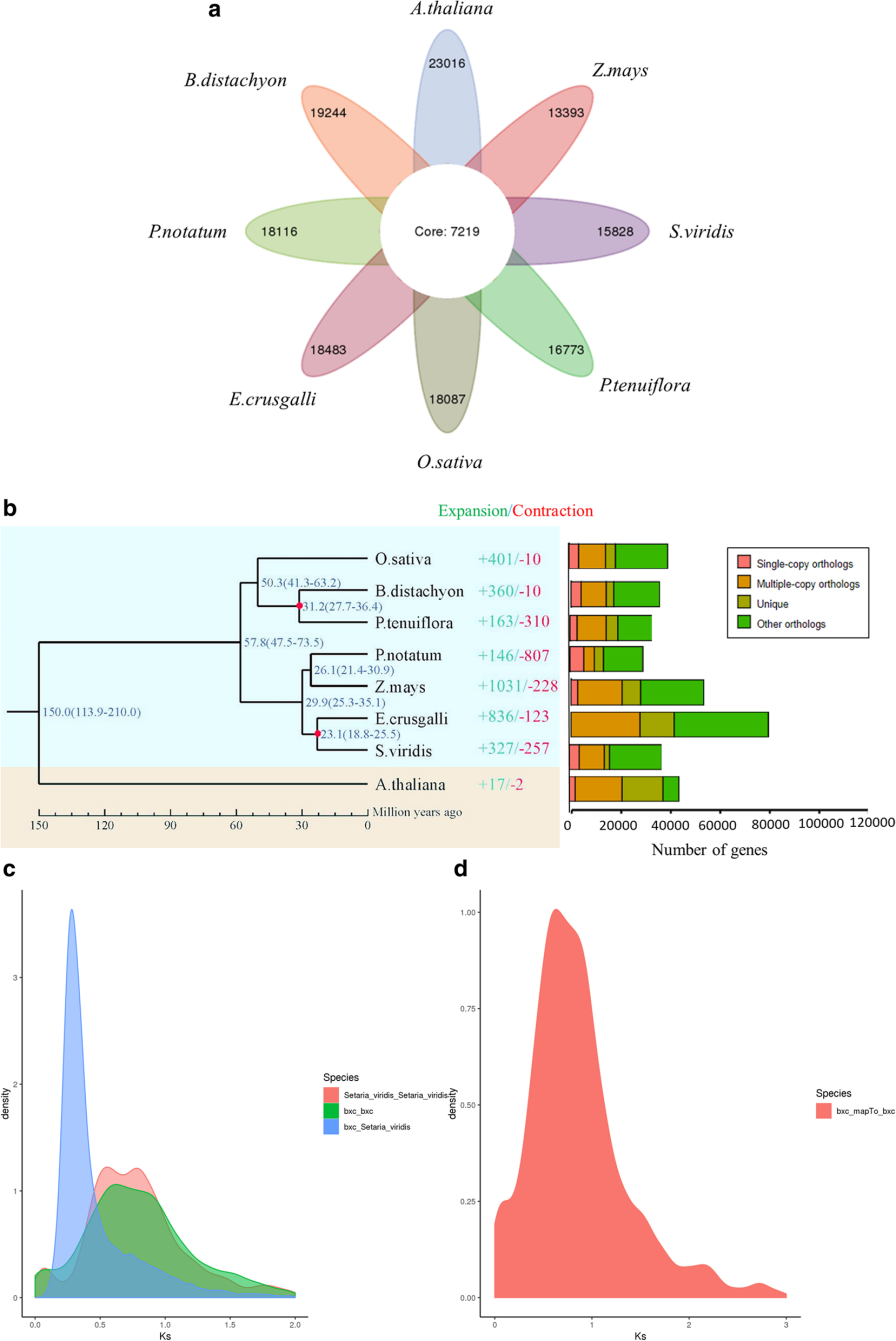
Gene family and phylogenetic tree analyses of *P. notatum* ‘Flugge’ and other representative plant genomes. **a** Venn diagram of the number of shared gene families. **b** A phylogenetic tree based on shared single-copy gene families (left), gene family expansions and contractions among *P. notatum* ‘Flugge’ and seven other species (middle), and Gene family clustering in *P. notatum* ‘Flugge’ and seven other plant genomes (right). **c** Genome-wide replication Ks distribution map of *P. notatum* ‘Flugge’ and its related species. **d** Genome-wide replication Ks analysis of *P. notatum* ‘Flugge’

A comparison of *P. notatum* ‘Flugge’ with the genomes of the six representative species combined with gene family analysis revealed that the 36,511 genes of *Paspalum notatum* ‘Flugge’ clustered with 25,335 gene families. The maximum number of clusters in Arabidopsis was 30,235. However, all the species included in the analysis shared 7219 gene families (Fig. 4a). The analysis suggested that *P. notatum* ‘Flugge’ expanded 146 gene families and contracted 807 gene families in the evolution process. GO analysis showed that the expanded gene family types were related to organic-inorganic compound synthesis, DNA biosynthesis, and nucleosides. Notably, gene families related to acid metabolism were the most enriched (Table S1). The gene families were potentially involved in plant growth metabolism and stress resistance, thus conferring *P. notatum* ‘Flugge’ with strong resistance, fast growth, and strong reproductive ability [37]. A phylogenetic tree was constructed using 5583 single-copy homologous genes, with *Arabidopsis thaliana* (TAIR10.1 from NCBI) [38] as the out-group. *P. notatum* ‘Flugge’*, Zea mays* (v5.0 from NCBI) [39], *Echinochloa crusgalli* (v2.0 from Bioinplant Lab of Zhejiang University) [40], and *Setaria viridis* (v2.0 from NCBI) [41] clustered together to form a monophyletic group [42]. *Zea mays* (maize) was more closely related to *P. notatum* ‘Flugge’ than the other species, with an estimation that it diverged about 26.1 million years ago (Fig. 4b).

The WGD events are important indices in plant evolution and are thought to be a driving force for plant adaptation to various environments [43, 44]. Changes in the synonymous replacement rate between paralogous genes were used to measure the duplication and loss of genes in the *P. notatum* ‘Flugge’ genome to explore its evolutionary history during the evolution process. The resultant data suggested that the differentiation of *P. notatum* ‘Flugge’ and *Setaria viridis* occurred before the WGD events. Both *P. notatum* ‘Flugge’ and *Setaria viridis* experienced a common WGD event when the KS value was 0.32 (Fig. 4c). In addition, the WGD event also occurred when the KS value of *P. notatum* ‘Flugge’ was 0.7 (Fig. 4d).

## Discussion

The gene and genome data of gramineous plants with excellent agronomic traits are an important resource for comparative genomics and functional omics. Paspalum is an excellent turfgrass whose high-quality chromosome-scale-based genome was assembled for the first time in this study. These findings improve the genomic resource library of gramineous plants and provide an excellent reference for future research on other Paspalum crops. The latest PacBio third-generation HiFi assembly and sequencing revealed that the genome size of *P. notatum* ‘Flugge’ was 541 M. The assembly result is the higher index among the genomes of grasses published so far, with a contig N50 = 52Mbp, scaffold N50 = 49Mbp, and BUSCOs = 98.1%, accounting for 98.5% of the estimated genome. Notably, the coverage of the assembled genome at the chromosome level was also very high (95.15%) after combining high-throughput sequencing and Hi-C scaffolding. Genome annotation revealed 36,511 high-confidence gene models, thus providing an important resource for future molecular breeding and evolutionary research.

*P. notatum* ‘Flugge’ belongs to the Poaceae family, with limited data regarding its performance in evolutionary history. Genome collinearity analysis revealed that *P. notatum* ‘Flugge’ and *Zea mays* had a good degree of genome collinearity. Both species belong to the Subtrib. Panicinae Reichb and are thus close in phylogeny and genetic relationship. Phylogenetic analyses revealed that *P. notatum* ‘Flugge’ diverged after *Oryza sativa*, *Puccinellia tenuiflora*, *Brachypodium distachyon*, and before *Setaria viridis* and *Echinochloa crusgalli*. These species share the same ancestor with *P. notatum* ‘Flugge’. The genome information of *P. notatum* ‘Flugge’ will help clarify the evolutionary process of gramineous species and provide a preliminary understanding of their evolutionary state. *P. notatum* ‘Flugge’ has good resistance to various stresses and can thus provide important genetic resources against biotic and abiotic stresses for Poaceae crops.

## Conclusion

This study is the first to report the high-quality chromosome-scale-based genome of *P. notatum* ‘Flugge’ assembled using the latest PacBio third-generation HiFi sequencing reads. The genome has a high coverage rate and the higher completeness index among the gramineous genomes that have been published to date. This study provides an excellent genetic resource bank for gramineous crops and crucial perspectives regarding the evolution of gramineous plants.

### Experimental procedures

For sequencing of genomic DNA, the sample was collected by a qualified postgraduate in vacuutainer tube, from the well-growing *P. notatum* ‘Flugge’ (2n = 20), planted in a light incubator in the Grassland Agri-husbandry Research Center. The standard plant followed ethics normswere and complies with Chinese and international regulations.

### DNA isolation and sequencing

*P. notatum* ‘Flugge’ cv. Crowver was selected as the sampling plant. The plant was grown in an incubator at the Qingdao Agricultural University in Shandong, China. Its leaves were sampled in liquid nitrogen followed by genomic DNA extraction using the Tiangen DNA secure kit. Sequencing of the DNA was done by Berry Hekang (Beijing, China) using the PacBio third-generation HiFi assembly sequencing platform. Quality and quantity control of the DNA samples were first done, followed by library preparation of the processed DNA, and the libraries were subjected to PE sequencing using Illumina NovaSeq. Reads containing adapters, duplicates, and a low sequence quality were first filtered, followed by a random selection of 10,000 of the reads for comparison with the NT library using the BLAST tool. There was no significant external contamination detected. Notably, K-mer analysis was performed to estimate the gene size, heterozygosity, and duplication ratio to have a general understanding of the genome in advance.

### Genome assembly and quality evaluation

The NanoDrop 2000 spectrophotometer was used to detect the quality of the genomic DNA [45, 46]. The purified genome was subsequently constructed into a SMRTbell library and then sequenced using the Pacbio SMRT technology [32]. The size of the library was detected using Agilent 2100 bioanalyzer. The obtained data was filtered and then processed using the smrtlink software for ccs processing [47–50]. The hifiasm software was used for assembly, followed by de-hybridization of the contig sequence using the purge-dups software [51, 52]. A single-copy orthologous gene library combined using tblastn, augustus, and hmmer software were finally used to evaluate the integrity of the assembled genome [33, 34, 53–56].

### Hi-C data analysis and chromosome construction

*Paspalum notatum* ‘Flugge’ leaf tissue (100 mg) was soaked in paraformaldehyde, a cell cross-linking agent, for 15 min to bind DNA. Glycine was then added to the mixture to terminate the chromatin cross-linking reaction, followed by collection and freezing of the treated tissues in liquid nitrogen. The tissues were then ground to powder to extract DNA. Biotin-labeled oligonucleotide ends were added during the end repair, and a covaris breaker was subsequently used to break the extracted DNA recovered into 350 bp fragments [57]. The DNA bound to biotin was then captured and purified using avidin magnetic beads, followed by library construction and sequencing using the Illumina PE150 platform [35]. The raw reads were filtered, followed by a random selection of 10,000 sequencing reads for comparison to the NT library using the BLAST tool to check for cell contamination [52, 58]. The JUICER software was then employed to compare the Hi-C data with the draft genome [34]. The 3D-DNA comparison was subsequently used to analyze the Hi-C library results to obtain valid Hi-C data and generate the chromosome level scaffold of the *P. notatum* ‘Flugge’ genome [59–61].

### Genome functional annotation

The RepeatMasker, MITE Hunter, LTRharvest, LTR Finder, LTR retriever, and RepeatModeler software were employed to analyze and predict the repetitive sequences to identify the MITEs and LTR transposable elements following the structure prediction method [62, 63]. The software parameters of LTRharvest and LTR Finder were -similar 90 -vic 10 -seed 20 -seqids yes -minlenltr 100 -maxlenltr 7000 -mintsd 4 -maxtsd 6 -motif TGCA -motifmis And -D 15000 -d 1000 -L 7000 -l 100 -p 20 -C -M 0.9 [64, 65]. The parameters of the RepeatModeler software used to identify the repetitive sequences in the masked genome from scratch were -engine ncbi -pa 60. In the same line, the parameters of the RepeatMasker software used to mask the repetitive sequences in the genome were -s -nolow -norna -gff -engine ncbi -parallel 20 [66].

The tRNAscan-SE software was used to predict the tRNA ab initio rRNA. Other types of ncRNA were searched using the Rfam database. Their specific information was obtained through similarity comparison [67–69].

All repetitive regions except tandem repeats were soft-masked for protein-coding gene annotation. The coding sequences of *Puccinellia tenuiflora* (v1.0 from BIGD) [70], *Zea mays* (v5.0 from NCBI) [39], *Sorghum bicolor* (NCBIv3 from NCBI) [71], *Brachypodium distachyon* (v3.0 from NCBI) [72] and *Echinochloa crusgalli* (v2.0 from Bioinplant Lab of Zhejiang University) [40] were downloaded. These coding sequences were subjected to Blast (v. 2.2.20) searches against the *P. notatum* ‘Flugge’ genome. Homologs containing premature stop codons and frameshifts were discarded. *P. notatum* ‘Flugge’-leaf RNA-seq data were aligned to *P. notatum* ‘Flugge’ contigs using GeMoMa-1.6.1 [73] and a comprehensive transcriptome database was built using PASA (v. 2.0.1) [74, 75]. Open reading frames were predicted using PASA (v. 2.0.1) and the resulting database was used to train parameters for the following four de novo gene prediction software packages: AUGUSTUS (v. 3.2.2), GeneMarker-ET (v. 4.57) [76], GlimmerHMM (v. 3.0.2), and SNAP. Predictions obtained using these packages were then combined using EVM, then 36,511 genes were retrieved and functionally annotated by blast searches against databases including NR, Swiss-Pro, eggNOG, GO and KEGG. Venn analysis of the five major databases was then performed to obtain more accurate gene functional annotation information.

### Comparative analysis

The Mummer software set at nucmer -g 1000 -c 90 -l 200 was employed to perform genome collinearity analysis on *P. notatum* ‘Flugge’ and its relative species [77, 78], *Zea mays,* to derive its evolution history. Notably, the OrthoMCL cluster analysis was used to identify the 8 gene protein families(*Z.mays*, *B.distachyon*, *S.viridis*, *O.sativa*, *A.thaliana*, *P.tenuiflora*, *E.crusgalli*) [79]. An all-vs-all BLAST alignment of all *P. notatum* ‘Flugge’ gene protein-coding sequences (with 1e-5 as the default e-value) was first performed [80], followed by a calculation of the sequence similarity. The Markov clustering algorithm was then used for cluster analysis (expansion coefficient is 1.5) to obtain the protein family clustering results. Single-copy genes of each species were selected as reference markers, and four-fold degenerate sites were used to construct supergenes because of the imperfect evolutionary research of *P. notatum* ‘Flugge’. The Mafft software was subsequently used for multiple sequence comparisons of supergenes. A suitable base substitution model was selected, followed by constructing a species-based maximum likelihood (ML) phylogenetic tree and estimating its differentiation time using the RAxML software. The mcmctree tool in the PAML software package (parameters: burn-in = 5,000,000, sample-number = 1,000,000, sample-frequency = 50) was used to estimate differentiation time based on the single-copy gene family [81]. The time calibration point (correction point) was derived from the Timetree website. The Cafe software was subsequently used to analyze the gene families changes between species and then perform a GO functional enrichment analysis on the gene families. The Branch-site model analysis method was employed to detect the positive selection occurring in a specific clade and only affects some sites. A research of *P. notatum* ‘Flugge’ and its related species was performed to select one-to-one orthology proteins, which were subsequently aligned using the PRANK software set at default. The Gblocks software set at -t = c -e = .ft. -b4 = 5 -d = y [82, 83], was then used to filter the alignment results. The CODEML test in PAML was then used to test the positive selection located in a specific branch and affecting certain sites only. The Chi2 program set at a degree of freedom = 2 in PAML was subsequently used to test the correction of multiple hypotheses [84].

The duplicate age distribution method was used to detect WGD events. Blastp was used to compare the longest protein sequence of genes in the genome of *P. notatum* ‘Flugge’. The MCScanX software was subsequently used to filter the comparison results [85], and the Yn00 tool in the PAML software package was used to calculate the synonymous replacement rate. A density distribution map based on the Ks values of all paralog gene pairs and Ks values of ortholog gene pairs between the genomes of *P. notatum* ‘Flugge’*, Setaria viridis*, and other related species was then drawn using Matlab [43].

## Supplementary Information

**Additional file 1.**

**Additional file 2.**

## Data Availability

All data generated and analyzed during this current study are available in the Grassland Agri-husbandry Research Center, Qingdao Agricultural University with permission from the Competent Authority. All sequencing data were submitted in NCBI Database having BioProject ID PRJNA789418 and details of software used are in Table S2. Biological materials used in this study available from the corresponding author.

## Abbreviations

NGS: Next-Generation Sequencing
CCS: Circular Consensus Sequencing
BUSCO: Benchmarking Universal Single-Copy Orthologs
Hi-C: High-through chromosome conformation capture
MITEs: Miniature inverted repeat transposable elements
LTR: Long terminal repeat
LTR-RT: Long terminal repeat retrotransposons
ncRNA: Non-coding RNA
NR: NCBI nucleotide sequences
GO: Gene Ontology
KEGG: Kyoto Encyclopedia of Genes and Genomes
WGD: Whole genome duplications
